# Microstructure Evolution and Shear Strength of the Cu/Au80Sn20/Cu Solder Joints with Multiple Reflow Temperatures

**DOI:** 10.3390/ma15030780

**Published:** 2022-01-20

**Authors:** Chaoyu Chen, Mingxu Sun, Zhi Cheng, Yao Liang

**Affiliations:** School of Materials Science and Engineering, Dalian Jiaotong University, Dalian 116028, China; chenchaoyu2001@outlook.com (C.C.); mingxus9627@sina.com (M.S.); liangyao@djtu.edu.cn (Y.L.)

**Keywords:** intermetallics, fracture, joining, microstructure, interfaces

## Abstract

In order to present the multiple reflow process during electronic packaging, the influence of the different short-time reheating temperatures on the microstructure and shear strength of the Cu/Au80Sn20/Cu solder joints was studied and discussed. The results showed that high-quality Cu/Au80Sn20/Cu solder joints were obtained with 30 °C for 3 min. The joints were mainly composed of the ζ-(Au,Cu)_5_Sn intermetallic compound (IMC) with an average thickness of 8 μm between Cu and solder matrix, and (ζ-(Au,Cu)_5_Sn +δ-(Au,Cu)Sn) eutectic structure in the solder matrix. With an increase in the multiple reflow temperature from 180 °C to 250 °C, the microstructure of the joint interface showed little change due to the barrier effect of the formed ζ IMC layer and the limitation of short-time reheating on the element diffusion. The eutectic structures in the solder matrix were coarsened and transformed from lamellar to the bulk morphology. The shear strength of the as-welded joint reached 31.5 MPa. The joint shear strength decreased slightly with reheating temperatures lower than 200 °C, while it decreased significantly (by about 10%) with reheating temperatures above 250 °C compared to the as-welded joint. The shear strength of the joints was determined by the brittle solder matrix, showing that the joint strength decreased with the coarsening of the δ phase in the eutectic structure.

## 1. Introduction

Miniaturization of electronic products promotes the development of ultra large scale (ULSI) and super large scale (SLSI) integrated circuits, which require a higher packaging density [1,2]. In recent years, new technologies such as System in Package (SiP), complemented by Multi-Chip Module (MCM) and 3D package, also known as Stacked Die Packaging (SDP), were proposed to solve the high-density packaging problem [3,4]. In the process, solders with different melting points were adopted in the successive manufacturing stages to prevent the previous solder joint from remelting. Therefore, selecting a high-temperature solder for the first stage plays a critical role in ensuring high joint performance.

With the popularity of Pb-free electronic products, Au-based solders have become the most suitable replacement solder for high-temperature soldering. The studies conducted by researchers such as W.D. Macdonald [5] and K. Bobzin [6] showed the high adaptability of the Au-based solder in bonding wafer-level packages (WLP) and chip-scale packages (CSP). Among them, the Au80Sn20 eutectic solder shows the advantages of high strength, excellent creep and fatigue resistance and the possibility of flux free soldering, resulting in its wide application in the field of the high-power electronic devices and optoelectronic packaging [7,8,9]. Considering that Cu is often applied as the substrate material, Cu/Au80Sn20 is a common preamble interconnection solder joint in electronic packaging.

The current research, involving Cu-Au80Sn20 joining, mainly focuses on the effect of aging on the microstructure and properties of the solder under service conditions. Wensheng Liu [10] investigated the interfacial microstructure evolution and shear behavior of Au-20Sn/(Sn)Cu solder joints after isothermal aging. The results showed that the AuSn_2_ and (Cu,Au)_6_Sn_5_ layers formed at the interface gradually disappeared and the interface configuration transformed from AuSn/AuSn_2_/(Cu,Au)_6_Sn_5_/Cu_3_Sn layers to AuSn/Cu_3_Sn layers. The shear strength of Au–20Sn/(Sn)Cu solder joints decreased continuously as the aging time increased. Chingyuan Hoa [11] studied the interfacial evolution and mechanical properties of Au-Sn solder jointed Cu heat sink during a high-temperature storage test. AuCu_3_ grows by consuming the AuCu compound by increasing high-temperature storage time. Moreover, the AuSn eutectic precipitations gradually decreased, which resulted in reduced hardness. Accordingly, reheating temperature and time have significant effects on the microstructure and properties of the solder joints. However, there are few reports on the effect of subsequent short-time reheating (soldering) cycles, from subsequent solder processes, on the microstructure and properties of first high-temperature solder joints.

In the current research, the reflow temperatures of three main types of solder were selected as reheating temperatures: In-based and Bi-based solder with joining temperatures below 180 °C [12,13], In-containing solder in 200~210 °C [14,15,16] and Pb-free Sn-Ag-Cu solder in 230~250 °C [17,18,19]. To simulate the effects of different subsequent low-temperature soldering on the microstructure and properties of previous high-temperature solder joints, the Cu substrate was soldered with Au80Sn20 solder in an air furnace. Then the joints were short-time reheated with different temperatures corresponding to the above three types of solder, namely 180 °C, 210 °C, and 250 °C. The microstructure evolution and shear properties of the joints were analyzed to investigate the effect of the secondary thermal cycle on the Cu/Au80Sn20/Cu as-welded joints.

## 2. Materials and Methods

**Figure 1:**
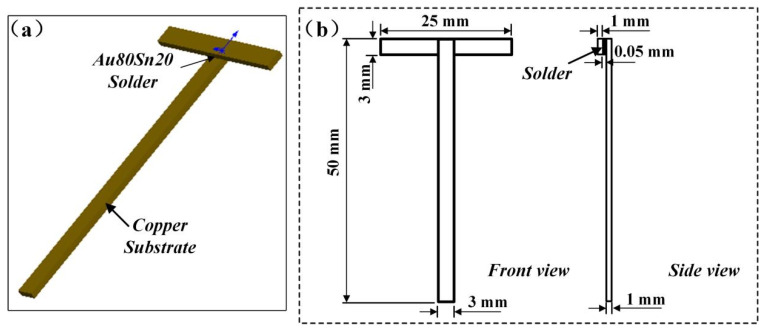
Shape and size of specimens. (**a**) Schematic diagram of welding assembly; (**b**) shape and size of specimens.

Pure copper of 1 mm thickness and Au80Sn20 (20 wt%) solder sheets of 0.05 mm thickness were used as the substrate and solder, respectively. The pure copper sheet was assembled into a T-joint, and the shape and size of the specimen are shown in Figure 1. The solder sheet was sandwiched between the overlapped two copper substrates, which is indicated by the arrow in Figure 1. A schematic diagram of the welding assembly is shown in Figure 1. Rosin mildly activated flux was selected to improve the wettability of the solder by removing the oxide film. During the welding process, the sample was quickly heated to 330 °C in an air furnace and held for 3 min, and then was air-cooled to room temperature. Finally, short-time reheating treatments of 180 °C × 3 min, 210 °C × 3 min, and 250 °C × 3 min were carried out on the as-welded joints to simulate the welding thermal cycles of the three types of the low-temperature solders.

**Figure 2:**
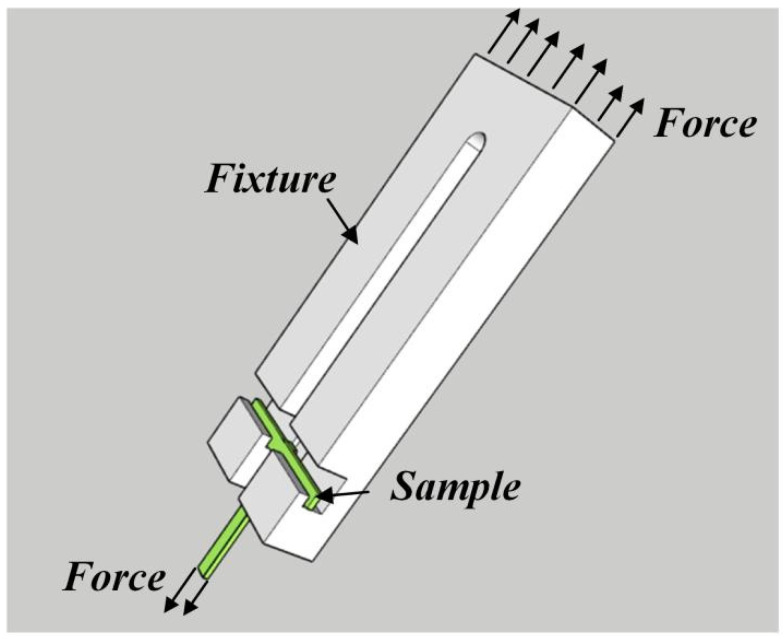
Schematic diagram of shear strength testing device.

After the welding and reheating treatment, the samples were cut to a size of 5 mm × 5 mm from the center of all the specimens for macroscopic and microscopic observation. Every specimen was polished by abrasive papers from 150 to 2000 grit size in succession, followed by fine polishing with flannelette. Finally, the Gatan 697 ion polisher was used for ion etching at a voltage of 4 KeV for 30 min. A scanning electron microscope (SEM) (Supra55, Carl Zeiss AG, Oberkohen, Jena, Germany) equipped with an energy dispersive spectrometer (EDS) (Oxford Xmax, Oxford Instruments, Abinden, Oxfordshire, UK) was used to observe the samples and analyze the microstructures of the joints. The shear strength of joints was evaluated by a UTM5105 electronic universal testing machine (Shenzhen SUNS TECHNOLOGY STOCK CO., LTD., Shenzhen, China). The schematic diagram of the shear strength testing device is shown in Figure 2.

## 3. Results and Discussion

### 3.1. Microstructure Evolution

**Figure 3:**
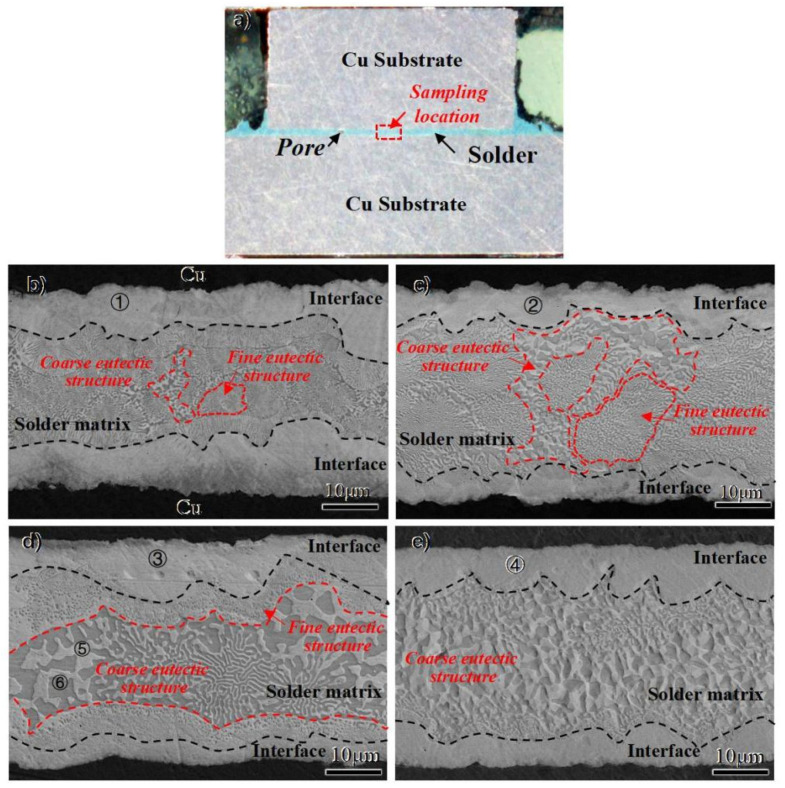
(**a**) Weld appearance of the as-welded joint; (**b**) microstructure of the as-welded joint; (**c**) microstructure of the 180 °C reheated joint; (**d**) microstructure of the 210 °C reheated joint; (**e**) microstructure of the 250 °C reheated joint.

The macrostructure of the as-welded joint is shown in Figure 3a. A joint with sound appearance was obtained at 330 °C for 3 min. Only a few pores existed in the solder matrix, which were attributed to the air condition during the welding process. Because the reheating temperature was below the liquidus temperature, the short-time reheating temperature showed little effect on the macrostructure of the joints. To evaluate the effect of the different multiple reflow temperatures on joint microstructure, the microstructures of the as-welded joint and joints with different reheating temperatures were analyzed and compared. As shown in Figure 3b–e, two regions with distinct morphologies are identified and denoted as interface and solder matrix, respectively. By comparing the different joints, the proportion of the interface and solder matrix regions shows little difference with the increase in the reheating temperature.

**Figure 4:**
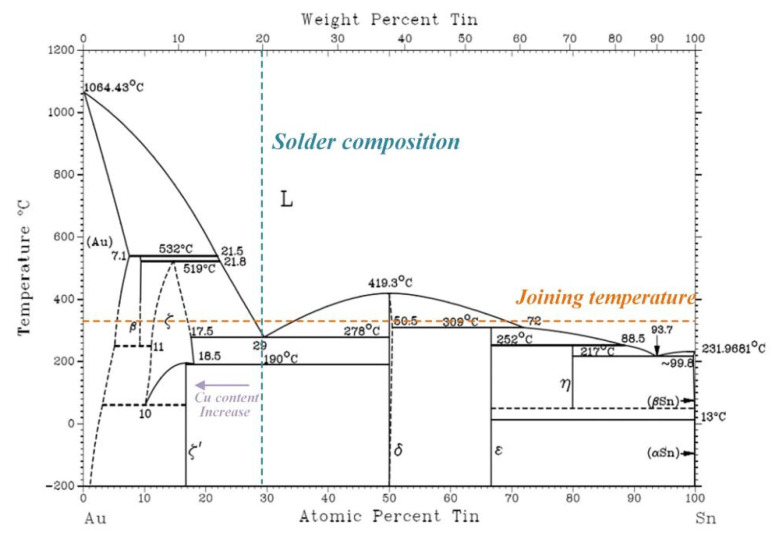
Au-Sn binary phase diagram.

**Figure 5:**
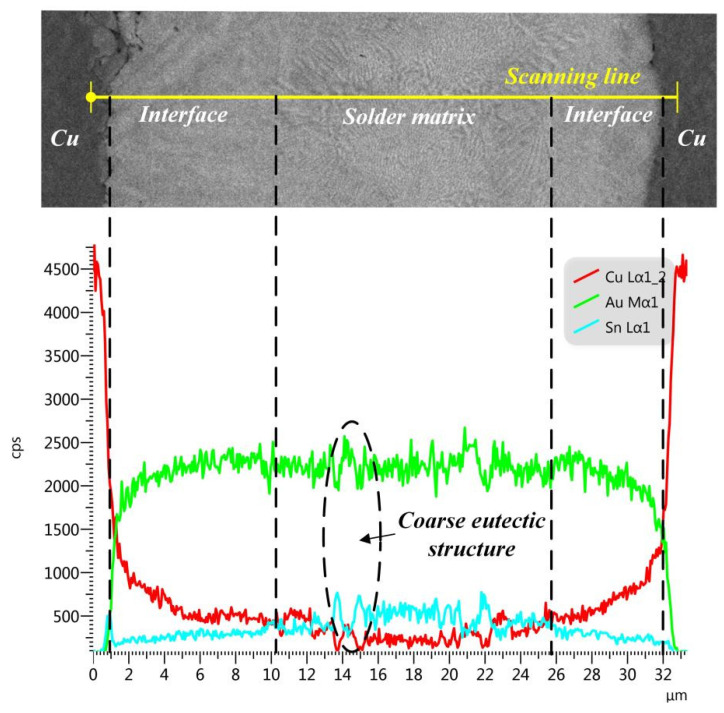
EDS line scanning of the as-welded joint.

In both the as-welded joints and reheated joints, an intermetallic compound (IMC) layer, with an average thickness of about 8 μm, was formed at the interface between Cu and solder matrix. The EDS analysis results are exhibited in Table 1. Combining the EDS results and Au-Sn binary phase diagram [20], as shown in Figure 4, it can be seen that all the interfaces are composed of the ζ-Au_5_Sn dissolved with many Cu elements (namely ζ-(Au, Cu)_5_Sn phase), which are respectively denoted by 1, 2, 3, and 4 in Figure 3b–e. Since Cu is chemically similar to Au, Cu atoms are likely to enter into the Au_5_Cu lattice and substitute for the Au atoms. With the increase in Cu content at the interface by the diffusing of Cu from the substrate, it was equal to the increase in Au content in Au-Sn binary phase and the ζ-Au5Sn phase with Cu participation formed, as shown in Figure 4. The phase was thus denoted as ζ-(Au, Cu)_5_Sn to show the Cu participation [21]. The EDS line scanning of the whole region in the as-welded joint is shown in Figure 5. At the interface, the Cu element exhibited typical diffusion characteristics that the element content decreased with the increase in distance from the Cu substrate. Furthermore, the decrease in the Cu element and the increase in the Au element at the interface was relatively stable.

By comparing the compositions, the constitution of the IMC at the interface between Cu and solder matrix exhibited little change despite the joints experiencing different reheating temperatures at 180~250 °C for a short time. This phenomenon indicated that the Cu element from the Cu substrate was dissolved into the liquid solder during the welding process. In contrast, the diffusion and migration of the Cu element was not remarkable during the short-time reheating below 250 °C. This can be attributed to the formed ζ phase hindering the diffusion of the Cu element from the Cu substrate to the solder. On the other hand, the short time and low multiple reflow temperature also limited the diffusion of the Cu element.

The eutectic structure in the solder matrix can be easily distinguished by the different morphologies compared to the ζ interface, as it was surrounded by the dense IMC layer on both sides. As shown in Figure 5, the Au80Sn20 solder liquefied at the joining temperature of 330 °C, then experienced the eutectic transformation: L-(ζ + δ), finally forming the (ζ + δ) eutectic structure. Two types of eutectic microstructures were found in the solder matrix: one with a fine lamellar microstructure and the other with a coarse microstructure, as shown in Figure 3b–e. The two structures exhibited different morphologies but the same composition, both of which comprised of (ζ + δ) phases, similar to previous studies [20]. Combined with the composition analysis and phase diagram, the brighter ones in the eutectic structure were the ζ-Au_5_Sn phase, while the darker was the δ-AuSn phase. The two phases also dissolved a certain amount of Cu, which were denoted by ζ-(Au, Cu)_5_Sn and δ-(Au, Cu)Sn, respectively. From the EDS line scanning result shown in Figure 4, the coarse lamellar structure can be easily found in the zone with the significant content fluctuation of the Cu and Sn elements.

Different from the interface, the microstructures of the solder matrix showed significant changes under different reheating cycles. Upon increasing the reheating temperature, the fine lamellar structure decreased while the coarse structure increased. This can be attributed to the reduction in free energy, caused by the reduction in interfacial energy decrease, promoting the aggregation and growth of the fine grains. After the short-time reheating treatment at 180–250 °C, the eutectic structure was coarsened with the increase in reheating temperature, and the lamellar structure gradually evolved to a bulk shape. When the reheating temperature reached 250 °C, the lamellar structures had almost disappeared, as shown in Figure 3e.

**Figure 6:**
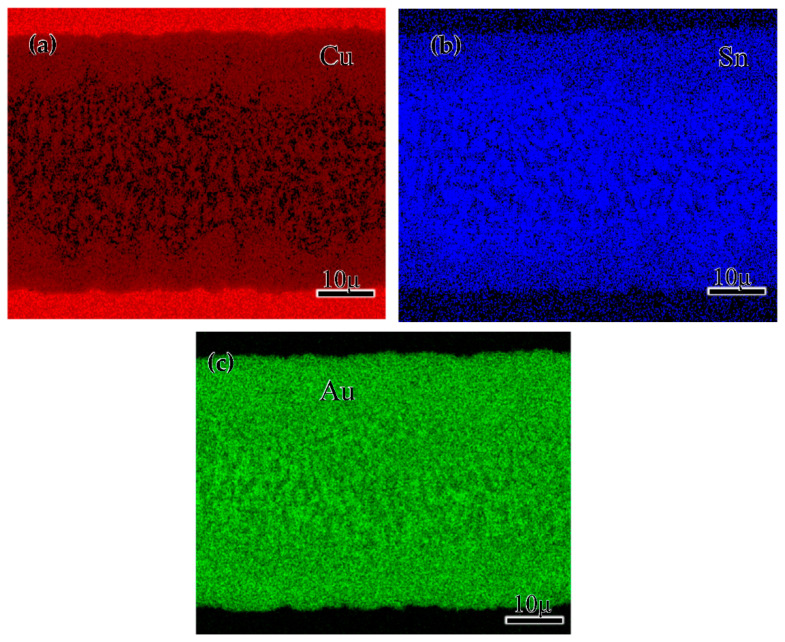
EDS element mapping results of the 250 °C reheated joint: (**a**) Cu element; (**b**) Sn element; (**c**) Au element.

Figure 6 shows the distribution of the Au, Sn, and Cu elements in the joints after the 250 °C reheating treatment. The Cu element from the substrate was mainly dissolved in the ζ phase, and the concentration of the Au element showed a slight fluctuation over the whole brazing seam. Meanwhile, Sn elements were mainly distributed in the δ phase at the middle of the solder matrix [21], which can also be proved by the results of phase components at points 5 and 6 in Figure 3 and Table 1. The formation enthalpy of the Au_5_Sn phase was lower than that of the AuSn, indicating that the Au_5_Sn preferred to form during the joining process. However, the binding energy of the Au_5_Sn was larger than other Au-Sn phases, indicating that its formation was unstable. Therefore, the Cu element preferred to dissolve in the Au_5_Sn and form the ζ-(Au, Cu)_5_Sn phase.

### 3.2. Shear Strength

**Figure 7:**
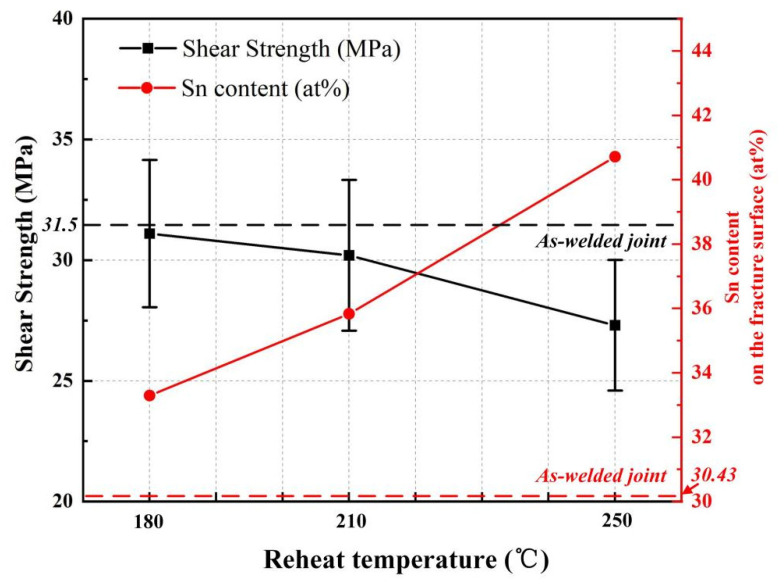
Shear strengths and the Sn content on the fracture surface of the as-welded joint and the joints with different reheating temperatures.

To evaluate the effect of the multiple reflow on the joint mechanical performance, the shear strength of the joints as welded and with different reheating temperatures were tested. The results are shown in Figure 7 and Table 2. The shear strength of the as-welded joint was 31.5 MPa, which was equivalent to that of the joint reheated at 180 °C. The shear strength decreased slightly after reheating treatment at 210 °C and decreased about 10% after reheating treatment at 250 °C. The short-time reheating treatment of the Cu/Au80Sn20/Cu solder joints below 200 °C showed little effect on the joint reliability. However, the shear strength of the reheating joints decreased when the temperature exceeded 200 °C, and decreased further with increasing reheat temperature. Many studies about joint high-temperature aging resistance showed that the brittle AuCu phase would form at the interface between the Cu matrix and ζ IMC layer when the Cu/Au80Sn20/Cu joints were exposed to a high-temperature environment for a long time [11,22]. Moreover, the shear strength of the joints rapidly decreased with the thickening of the AuCu IMC layer. However, from the microstructure analysis mentioned above, it can be seen that the AuCu phase was not formed during the short-time reheating treatment. This can be attributed to the inactive alloy element diffusion, especially the Cu element, under the short-time reheating cycle.

**Figure 8:**
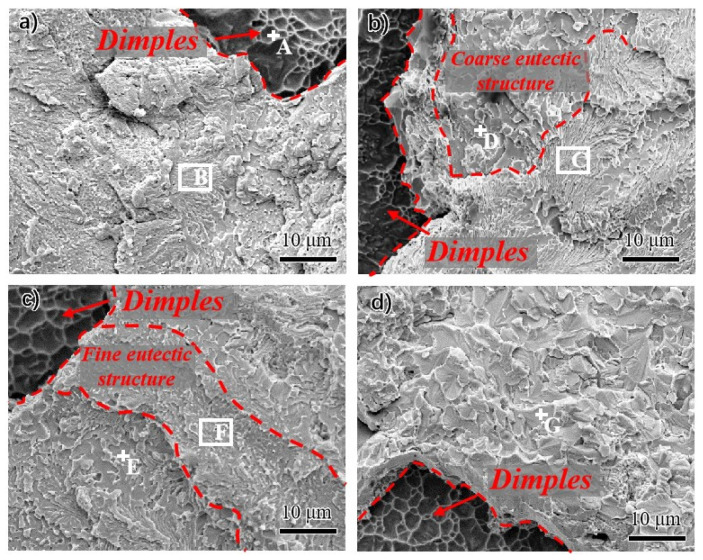
Fracture surface of the joints. (**a**) As-welded joint; (**b**) 180 °C reheated joint; (**c**) 210 °C reheated joint; (**d**) 250 °C reheated joint.

The fracture photograph of joints with different reheating temperatures was investigated by SEM, and the phase compositions in the fracture surface were tested by EDS. The microstructures of the fracture surface are shown in Figure 8, and the corresponding EDS spot and region test results are shown in Table 3. All samples exhibited mixed fracture features combined with ductile and brittle modes. A ductile fracture region with dimples was located at the interface between the Cu matrix and the solder. In contrast, the brittle fracture region with cleavage or quasi cleavage characteristics was located at the Au-Sn eutectic structure zone, as shown in Figure 8. According to the EDS results, Cu(s,s) was detected in the dimples, which resulted in the ductile fracture in this zone. The fracture position of the eutectic zone was carefully observed, and the EDS results are shown in Table 2. Consistent with the results of microstructure observation, the fine lamellar and coarse eutectic structures were observed on the brittle region, indicating that the eutectic structure of the solder matrix was the weak part under the shear tension. During the shear process, the cracks were initiated in the eutectic zone and then propagated to the interface between Cu and solder matrix. Meanwhile, the fracture surface also showed that the fine eutectic area decreased while the coarse eutectic area increased with the increase in the reheating temperature. Furthermore, the joints were all fractured in a transgranular form.

The influence of the multiple reflow temperatures on the joint shear strength and Sn content on the fracture surface is shown in Figure 7. The shear strength decreased gradually while the content of Sn increased slightly with the increasing reheat temperature. This phenomenon can be attributed to the high sensitivity of the low strength phase to crack initiation and propagation. The elastic modulus and shear modulus of Au_5_Sn is greater than that of AuSn; thus, the crack propagated preferentially from the AuSn phase in the eutectic region. In the joint with lamellar eutectic structure, the joints showed high shear resistance and strength due to the fine grain of the AuSn phase and the inhibition effect of adjacent high-performance phase Au_5_Sn on the cracks. However, the increase in AuSn phase size promoted the sensitivity of the crack initiation and propagation with the lamellar eutectic structure transformed into a coarse bulk eutectic structure, finally resulting in more AuSn phase appearing on the fracture surface. This phenomenon can be proved by the presence of the AuSn phase on the coarse eutectic structure on the fracture surface, as shown in Table 3.

## 4. Conclusions

(1) The Cu substrate was soldered with Au80Sn20 solder, and high-quality joining of the Cu/Au80Sn20/Cu was realized at a temperature of 330 °C for 3 min. The joints were mainly composed of ζ IMC with an average thickness of 8 μm between the Cu and the solder matrix, and (ζ +δ) eutectic structure in solder matrix.

(2) Under multiple different reflow temperatures, the microstructure of the joint interface showed little difference due to the barrier effect of the formed ζ IMC layer and the limitation of short-time reheating on the element diffusion. With the increase in the multiple reflow temperatures, the eutectic structures in the solder matrix were coarsened and transformed from lamellar to bulk structure.

(3) The shear strength of the as-welded joint reached 31.5 MPa. The joint shear strength decreased slightly with short-time reheating temperatures lower than 200 °C, while it decreased significantly when reheating temperatures reached 250 °C, showing a 10% decrease compared to that of the as-welded joint. The shear strength of the joints was determined by the brittle solder matrix, showing that the joint strength decreased with the coarsening of the δ phase in the eutectic structure.

## Figures and Tables

**Table 1:** Chemical composition of points 1–6 in Figure 3 and possible phases (at%).

	Au	Sn	Cu	Possible Phase
1	53.47	12.90	33.63	ζ
2	52.96	12.83	34.21	ζ
3	52.97	12.69	34.33	ζ
4	53.48	12.97	33.55	ζ
5	54.91	14.26	30.38	ζ
6	49.91	48.40	1.70	δ

**Table 2:** Shear strengths of the as-welded joint and the joints with different reheating temperatures.

Reheating Temperature (°C)	As-Welded	180	210	250
Shear strength (MPa)	31.5	31.1	30.2	27.3
Sn content (at%)	30.43	32.51	35.83	40.71

**Table 3:** Chemical compositions of the phases denoted by arrows in Figure 8 (at%).

	Au	Sn	Cu	Possible Phases
A	0.78	0.22	99.01	Cu(s,s)
B	55.32	30.43	14.26	(ζ + δ)
C	55.39	32.51	12.10	(ζ + δ)
D	50.33	43.61	6.06	δ
E	48.62	44.21	7.17	δ
F	52.30	35.83	11.88	(ζ + δ)
G	52.27	40.71	7.02	δ
